# Characterization of the complete mitogenomes of Baillon's Crake *Porzana pusilla* and phylogenetic analysis

**DOI:** 10.1080/23802359.2021.1875915

**Published:** 2021-02-12

**Authors:** Ting Lian, Chao Yang, Hao Yuan, Qing-Xiong Wang, Xiao-Juan Du, Xue-Juan Li

**Affiliations:** a Research Center for Prevention and Treatment of Respiratory Disease, School of Clinical Medicine, Xi’an Medical University, Xi’an, China; b Shaanxi Institute of Zoology, Xi’an, China; c School of Life Sciences, Shaanxi Normal University, Xi’an, China

**Keywords:** *Porzana pusilla*, mitochondrial genome, Rallidae, phylogeny

## Abstract

The complete mitochondrial genome of the Baillon's Crake *Porzana pusilla* (Gruiformes: Rallidae) are sequenced and annotated, which contained 37 typical genes. The length of the complete mitochondrial genome is 16,966 bp (GenBank No. MW043485), with As, Ts, Cs, Gs, and AT content of the mitochondrial genome is 32.1, 23.2, 30.9, 13.8, and 55.3%, respectively. All protein-coding genes started with ATN except *COX1* and *ND5*, which start with GTG, and all protein-coding genes end with a complete triplet codon (TAA, AGG, AGA, and TAG), except *COX3*, which ends with an incomplete T. The *ND3* gene of *P. pusilla* with an extra C nucleotide in 174 site. Phylogenetic analysis revealed that the new sequenced species of *P. pusilla* was closer to the clade of *Porzana fusca* and *Porzana paykullii*, and all three *Porzana* are clustered into one branch.

Rallidae birds include approximately 40 genera and 143 species, and most of them dwell on dense ground vegetation which is very difficult to observe in the field (Steadman 2000; Ruan et al. 2012; Chen et al. 2017). In the family of Rallidae birds, *Porzana* is the genus that contains the greatest number of species (García-R et al. 2014). The Baillon’s Crake *Porzana pusilla* belongs to the genus *Porzana* and the family Rallidae, is a very shy bird, which is found in marshes, along the edges of lakes and ponds and among paddy fields (Bhattacharjee 2012). Obtaining the complete Mitochondrial DNA sequence of *P. pusilla* is useful in further investigating this species.

Naturally dead *P. pusilla* was collected at Yulin, Shaanxi, China (38°3'49" N, 109°30'14" E), and the specimen (Proof number: XTJ01) was deposited in the animal specimens museum of Shaanxi Institute of Zoology, Xi’an, China. The complete mitochondrial genome of *P. pusilla* was sequenced using the Illumina HiSeq Xten platform (Illumina, CA, USA), and the genomic DNA was prepared with a paired-end (2 × 150) libraries. In totally, 7,225,062 paired-end raw reads were obtained. After removing regions with a Phred score of <10, the high-quality reads were then assembled into mitochondrial genome, and MITObim version 1.9 software (Hahn et al. 2013) was used with the complete mitogenome of *P. paykullii* (MG200164) as a reference. Finally, a total of 32,782 individual mitochondrial reads gave an average coverage of 296.3X. The assembled complete mitogenome of *P. pusilla* is 16,966** **bp in length (GenBank: MW043485), including 13 protein-coding genes (PCGs), 22 tRNA genes, 2 rRNA genes and 1 D-loop region. The 13 protein-coding genes were annotated using Genious v2020.0.5 by comparing to *P. paykullii*, and the 22 tRNA genes and two rRNA genes were predicted by the online MITOchondrial genome annotation Server (MITOS; http://mitos.bioinf.uni-leipzig.de/index.py). The base composition of *P. pusilla* mitogenome are as follows: A = 32.1%, T = 23.2%, C = 30.9%, G = 13.8%, and A + T = 55.3%. With the exception of *COX1* and *ND5*, which start with GTG, all protein-coding genes have typical ATN start codons, and all protein-coding genes end with a complete triplet codon (TAA, AGG, AGA, and TAG), except for *COX3*, which ends with an incomplete T.

Furthermore, in *ND3* gene of *P. pusilla* mtDNA, an extra nucleotide ‘C’ was present in 174 site, which is consistently observed with most species in Gruiformes. Someone considered that this extra nucleotide would be cut down by RNA editing during translation, the function of *ND3* gene would be recovered and avoided effectively the premature stopping of transcription due to frameshift mutation (Mindell et al. 1998). The latest hypotheses was that the ability to tolerate frameshift insertions was implicated in certain mitochondrial translation systems by using programmed translational frameshifting (Russell and Beckenbach 2008). The function of the extra ‘C’ in *ND3* gene and its phylogenetic significance are worthy of further scrutiny.

The length of 22 tRNA genes ranked from 67** **bp (*tRNA-Ser*(AGY) and *tRNA-Cys*) to 77** **bp (*rRNA-Leu*(UUR)), and all tRNA genes have the typical cloverleaf secondary structures, with the exception of *tRNA-Ser*(AGY), which the dihydrouridine arm forms a simple loop. The length of 12S rRNA was 974** **bp and 16S rRNA was 1588** **bp, located between *tRNA-Phe* and *rRNA-Leu*(UUR), separated by *tRNA-Val*. The D-loop region was 1453** **bp in length, located between *tRNA-Glu* and *tRNA-Phe*, and the AT content of the D-loop region was 57.2%.

To validate the phylogenetic position of *P. pusilla*, a phylogenetic analysis was performed on the concatenated datasets of 13 PCGs and 2 rRNAs of the newly sequenced mitochondrial genome and 20 Rallidae birds downloaded from GenBank, with one *Grus japonensis* (Family: Gruidae) selected as outgroup. Alignment of all 13 PCGs inferred from the amino acid alignment were performed using MEGA v7.0 (Kumar et al. 2016), and the alignment results were then concatenated using SequenceMatrix v1.8 (Vaidya et al. 2011). PartitionFinder v2.1.1 (Lanfear et al. 2012) was used to determine the best partitioned scheme and the optional model of evolution. Maximum likelihood tree was constructed using IQ-TREE (Nguyen et al. 2015) with 5000 bootstrap replicates to estimate branch support. The topology was given, and Branch support test numbers and bootstrap support numbers are shown in Figure 1. Within phylogenetic tree, *P. pusilla* as the sister group of the clade of *P. fusca* and *P. paykullii* with strongly supported, and then all three *Porzana* are clustered into one branch.

**Figure 1.**
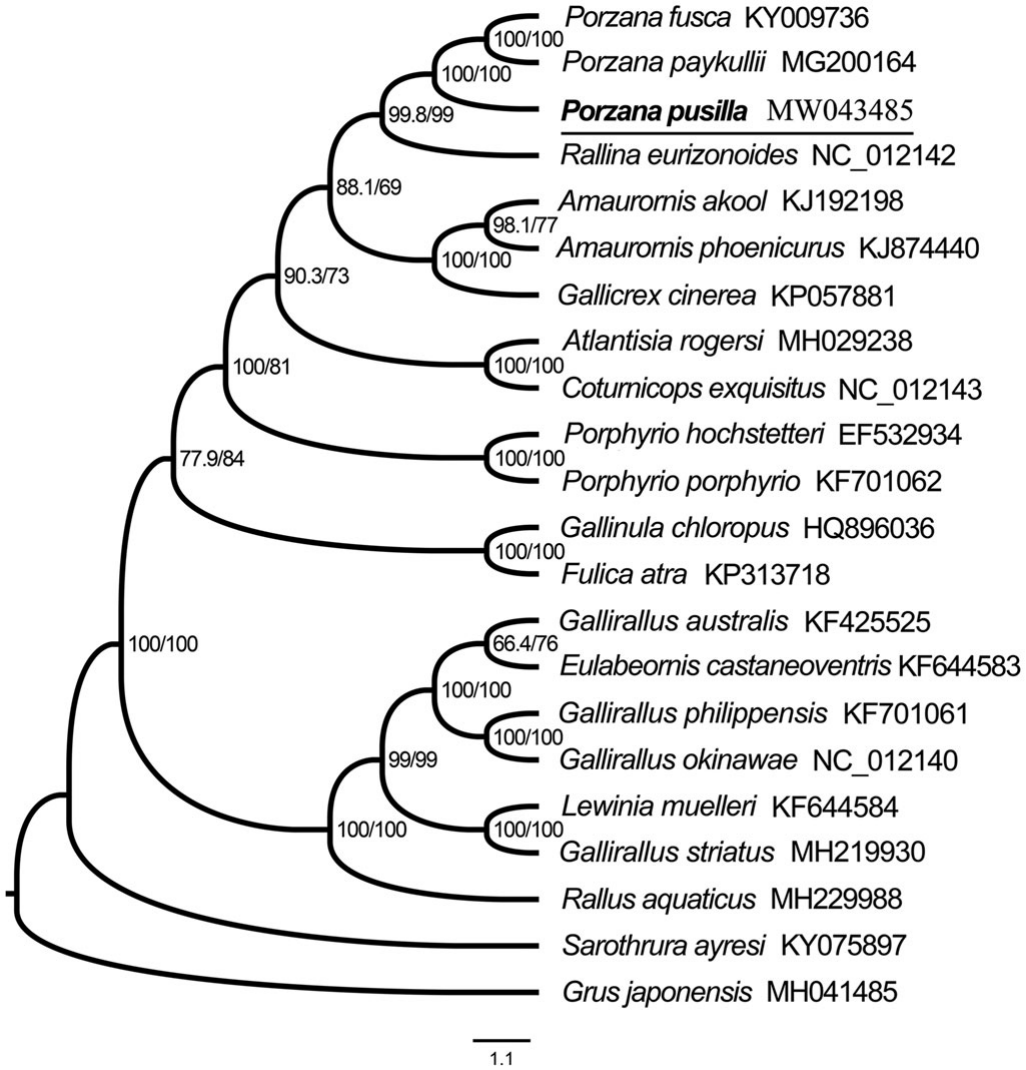
Maximum likelihood phylogenetic tree of 21 Rallidae birds and one outgroup based on 13 PCGs and two rRNAs under the software IQ-TREE. All species sequences except for *P. pusilla* were downloaded in GenBank, and the accession number was given with species names. (Node supports from left to right are SH-aLRT Single branch test value and bootstrap value).

## Data Availability

The data that support the findings of this study are openly available in NCBI at https://www.ncbi.nlm.nih.gov/, reference number [MW043485], or available from the corresponding author.
